# Supplementary material for: In situ single-shot diffractive fluence mapping for X-ray free-electron laser pulses
Source: Nat Commun. 2018 Jan 15;9:214. doi: 10.1038/s41467-017-02567-0 (PMC5768711; doi:10.1038/s41467-017-02567-0)
Supplement: Supplementary file 1 — Supplementary Information [file 41467_2017_2567_MOESM1_ESM.pdf]

Supplementary Note 1: Relationship to Fresnel zone-plates

The grating formula we derive in the main text,

$$t(\xi,\eta) = \frac{1}{2} + \frac{1}{2}\cos\left(\frac{2\pi}{p(\xi,\eta)}\left(\xi\cos\left(\varphi(\xi,\eta)\right) + \eta\sin(\varphi(\xi,\eta))\right)\right) \tag{1}$$

with

$$\varphi(\xi,\eta) = \arctan\left(\frac{y_0 + m\eta}{x_0 + m\xi}\right) \quad \text{, and} \tag{2}$$

$$p(\xi,\eta) = \frac{\lambda}{\sin\left(\arctan\left(\sqrt{(x_0+m\xi)^2 + (y_0+m\eta)^2}/z_{\mathrm{det}}\right)\right)} \tag{3}$$

describes segments of Fresnel zone plates. This can readily be verified by inspecting gratings whose diffraction center is coaxial with the illuminating beam, i.e. by setting $x_0 = y_0 = 0$. The radius of the n-th zone of an equivalent zone-plate is [1]

$$r_n = \sqrt{n\lambda f}\,. \tag{4}$$

Where the focal distance f is related to our grating parameters by

$$f = \frac{z_{\mathrm{det}}}{2m}\,. \tag{5}$$

Leveraging these parameters, we can ensure that the focal distance does not exceed the Rayleigh-length, given by

$$z_{\mathrm{R}} = \frac{\pi w_0^2}{\lambda}\,. \tag{6}$$

Demanding that $f < z_{\mathrm{R}}$ directly yields the condition for the grating design we use in the main text:

$$\frac{z_{\mathrm{det}}}{m} < 2\pi\frac{w_0^2}{\lambda} \tag{7}$$

This result has a very illustrative interpretation: Since Supplementary Equation (7) forbids to increase the detector distance arbitrarily, smaller grating structures are necessary to achieve sufficiently large deflection angles to separate the fluence map from the main beam. This ensures that the grating features are always considerably smaller than the beam footprint, and thus, that the illumination does not change drastically within a small number of grating periods. Such a limit is to be expected, since our derivation of the grating formula starts with the assumption of a well-defined diffraction spot from a regular grating, which requires a minimum number of illuminated grating periods.

The remaining grating parameters (x_0, y_0), i.e. the center of the fluence map on the detector, have no influence on the equivalent zone-plate parameters. Instead, they determine – in conjunction with z_{det} and m – which segment of the zone-plate constitutes the final grating sample.

Supplementary Note 2: Simulations of the beam footprint

In order to decide whether a particular grating is capable of mapping a certain spot, we consider the size of the focal spot in relation to the spatial structure size of the grating. We simulate a grating with $\lambda = 20.8\,\mathrm{nm}$, $z_{\mathrm{det}} = 12\,\mathrm{cm}$, $x_0 = y_0 = 7\,\mathrm{mm}$ and $m = 500$. These parameters translate to a grating half-pitch of

$$\frac{p}{2} = \frac{\lambda}{2\cdot\sin(\arctan(\sqrt{x_0^2 + y_0^2}/z_{\mathrm{det}}))} = 110\,\mathrm{nm} \quad . \tag{8}$$

The illumination is a plane wave with a circular shape. We modify the steepness of the illumination profile by convolving it with a Gaussian profile. The full width at half maximum (FWHM) of this Gaussian is given – in multiples of the grating's structure size – on top of the respective images in Supplementary Figure 1a–d. We simulate the diffraction pattern by calculating the Fourier transform of the product of grating and illumination function (Supplementary Figure 1e–h).

It is evident, that the diffracted fluence map resembles the actual illumination more closely for larger widths of the Gaussian smoothing. The line-scans in the simulated example (Supplementary Figure 1i–l) show good agreement when the illumination change spans at least 5 grating periods ($N = 10$) or more.

We can derive this rule-of-thumb by considering under what circumstances two spots of width s and at lateral distance $(1+\epsilon)s$ will still be distinguishable in the diffracted fluence map. On the detector, all sizes and distances are scaled by the magnification factor m, according to the grating design. Additionally, the spots are smeared out by the width of the principal maximum of a grating with finite number of illuminated lines. With the grating periodicity p and the number of illuminated lines N, we get

$$(1+\epsilon)ms \geq ms + \frac{z_{\text{det}}\lambda}{Np} \quad \text{, or} \tag{9}$$

$$\epsilon ms \geq \frac{z_{\text{det}}\lambda}{Np} \quad . \tag{10}$$

In small-angle approximation, p is given by:

$$p \approx \frac{z_{\text{det}}\lambda}{r} \quad \text{, with} \tag{11}$$

$$r = \sqrt{(x_0 + m\xi)^2 + (y_0 + m\eta)^2} \quad . \tag{12}$$

And thus

$$N \geq \frac{r}{\epsilon ms} \tag{13}$$

Since the number of illuminated lines is $N = s/p$, we arrive at

$$p \leq \frac{ms^2}{\epsilon r} \tag{14}$$

which, for the above example with grating periods of 220 nm and $\epsilon = 1$, is satisfied when $s \geq 1.8\,\mu\text{m}$. This matches our observations from the simulated diffraction patterns. We note that this is only a rough estimate that, for example, depends heavily on what distance is considered distinguishable.

When we express Supplementary Equation (14) in terms of the detector distance (i.e. by again substituting $p = \lambda z_{\text{det}}/r$), we get an expression that is very similar to Supplementary Equation (7), demonstrating that both perspectives are equivalent:

$$\frac{z_{\text{det}}}{m} \leq \frac{1}{\epsilon}\frac{s^2}{\lambda} \tag{15}$$

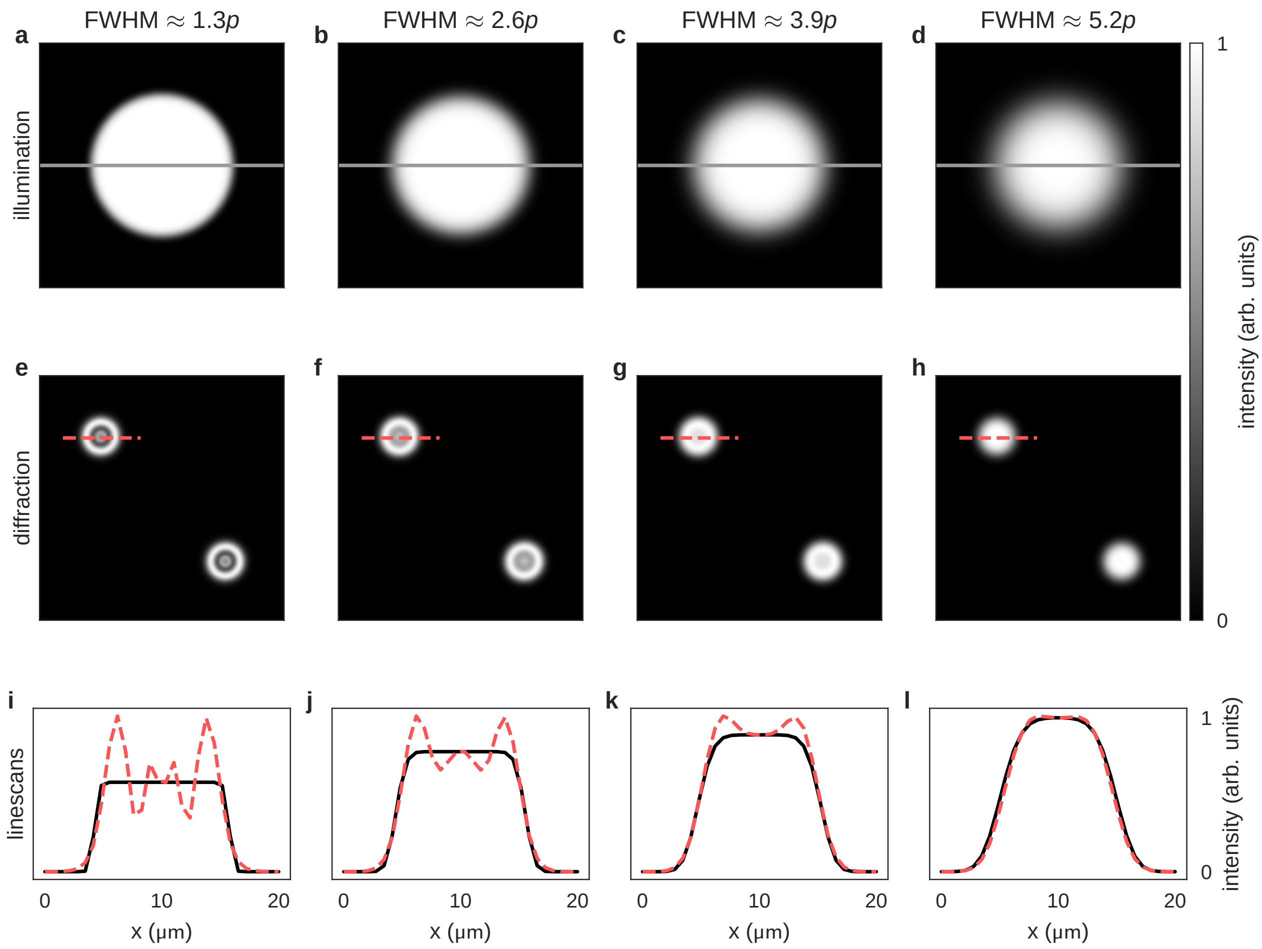

Supplementary Figure 1: **Simulated diffraction signals of increasingly soft illumination profiles. a–d)** Plane-wave illumination profiles with circular shape. The borders of the illumination functions have a Gaussian profile with a full width at half maximum (FWHM) of (a) 0.5 µm, (b) 0.9 µm, (c) 1.4 µm and (d) 1.9 µm. These roughly correspond to 1.3, 2.6, 3.9 and 5.2 times the grating period p, respectively. **e–h)** Simulated Fraunhofer diffraction. The high-intensity center pixel that corresponds to the undeflected beam ($q = 0$) is masked for better visibility of the diffraction. For steep illumination gradients, i.e. when the intensity changes strongly within less than 5 grating periods (ten times the half-pitch), the mapped fluence distribution deviates from the actual illumination. **i–l)** Line profiles of the illumination (black solid lines) and the diffracted fluence map (red dashed lines). Corresponding lines mark the respective positions in the illumination and diffraction images. The simulated sample and illumination are 10 µm × 10 µm in size. The diffraction images are cropped to represent the available detector area of our 27.6 mm × 27.6 mm CCD camera at $z_{\text{det}} = 12$ cm distance.

Supplementary References

1. Thompson, A. C. *et al.* *X-ray Data Booklet* 2nd ed. (Center for X-ray Optics and Advanced Light Source, Berkeley, 2001).
